# Subcortico-Cortical Functional Connectivity in the Fetal Brain: A Cognitive Development Blueprint

**DOI:** 10.1093/texcom/tgaa008

**Published:** 2020-04-03

**Authors:** Matteo Canini, Paolo Cavoretto, Paola Scifo, Mirko Pozzoni, Alessandro Petrini, Antonella Iadanza, Silvia Pontesilli, Roberta Scotti, Massimo Candiani, Andrea Falini, Cristina Baldoli, Pasquale A Della Rosa

**Affiliations:** 1 Department of Neuroradiology, San Raffaele Scientific Institute, 20132 Milan, Italy; 2 Department of Gynecology, San Raffaele Scientific Institute, 20132 Milan, Italy; 3 Department of Nuclear Medicine, San Raffaele Scientific Institute, 20132 Milan, Italy; 4 Department of Computer Science, Università degli Studi Milano, 20122 Milan, Italy

**Keywords:** brain development, cognitive control, learning, resting state fMRI, sensorimotor

## Abstract

Recent evidence has shown that patterns of cortico-cortical functional synchronization are consistently traceable by the end of the third trimester of pregnancy. The involvement of subcortical structures in early functional and cognitive development has never been explicitly investigated, notwithstanding their pivotal role in different cognitive processes. We address this issue by exploring subcortico-cortical functional connectivity at rest in a group of normally developing fetuses between the 25th and 32nd weeks of gestation. Results show significant functional coupling between subcortical nuclei and cortical networks related to: (i) sensorimotor processing, (ii) decision making, and (iii) learning capabilities. This functional maturation framework unearths a Cognitive Development Blueprint, according to which grounding cognitive skills are planned to develop with higher ontogenetic priority. Specifically, our evidence suggests that a newborn already possesses the ability to: (i) perceive the world and interact with it, (ii) create salient representations for the selection of adaptive behaviors, and (iii) store, retrieve, and evaluate the outcomes of interactions, in order to gradually improve adaptation to the extrauterine environment.

## Introduction

### Theoretical Background

Brain ontogeny can be fully characterized only through delineating how emerging brain structures start “connecting” throughout gestation. Cortical development in the perinatal and neonatal period is dramatically fast (Van Den Heuvel and Thomason 2016) and entails subtle morphological changes which are difficult (or not possible) to detect through conventional structural magnetic resonance imaging (MRI) (Conte et al., 2016). Nevertheless, assuming that changes in structural maturation likely exert a direct impact on brain activity, the study of functional connectivity may open an exclusive window for an in vivo observation of cerebral microstructural development. It is only very recently that a primal sketch of the developing functional connectome is taking shape (Van Den Heuvel et al. 2018; Turk et al. 2019) through new methodological approaches allowing to cope with the influence of fetal movements on functional activation patterns (Rutherford 2019). Evidence so far suggest that the functional fetal brain has a modular organization (Thomason et al. 2014) and that prototypical, adult-like resting state networks are consistently detectable in utero starting from as early as the beginning of the third trimester of pregnancy (Schöpf 2012). Early functional connectivity patterns are preferentially short-ranged and tend to remain segregated within a single hemisphere, with both distance and bilaterality increasing as a function of gestational age (Thomason et al. 2013, 2015). Maturation occurs progressively and follows a developmental gradient characterized by networks underlying the processing of raw, sensorimotor information synchronizing and reaching maturity before those supporting higher order cognition (Jakab 2014).

### Aim of the Work

Seminal evidence through in vivo and ex vivo investigations (Kostovic and Jovanov-Milosevic 2006; Kostovic and Judas 2010; Kostovic et al. 2019) has shown that thalamo-cortical afferentation occurring during the third trimester of pregnancy is an ontogenetic cornerstone, providing the neurophysiological infrastructure for the development of a sensory-expectant cortex. The subcortico-cortical developmental tie is a prerequisite for information gathered through senses to be processed and integrated into higher-order cognition and thus represents the foundational premise for newborn survival in the external environment. Cognitive development, in turn, relies on the synchronous activation of several areas to occur and is best detected and represented by means of functional investigation (Cao et al. 2014). Accordingly, functional patterns of intrinsic fetal cortical synchronization have been consistently detected during the last trimester of gestation (Thomason et al. 2013, 2014, 2015; Schöpf 2012; Jakab 2014). However, despite their pivotal role in cognition (Raznahan et al., 2014), the involvement of subcortical structures in early functional and cognitive development has never been explicitly investigated. We argue that linking: 1) patterns of subcortico-cortical functional connectivity observed during gestation to, 2) their future cognitive relevance, and, thus, 3) their ontogenetic significance will prove crucial to deepen our understanding of both normal cognitive development trajectories as well as of pathological outcomes throughout the lifespan.

To this aim, in this study we focus on the investigation of nascent patterns of functional connectivity between some major subcortical nuclei (i.e. the thalamus and subthalamic nucleus, the hippocampus, the amygdala, and the dorsal striatum [DS]) and the developing neocortex between the 25th and the 32nd week of gestation (GW). We assume observed patterns of functional subcortico-cortical connectivity to follow a developmental priority gradient through which functional networks underlying nuclear cognitive abilities are ontogentically planned to mature first. We thus seek for a “cognitive development blueprint”, through which a nuclear set of “cognitive tools” is trained during gestation and provided to the upcoming newborn in order to maximize the probability of adaption and survival in the extrauterine environment.

## Materials and Methods

### Participants

A sample of 22 fetuses recruited at San Raffaele Hospital in Milan, Italy, was considered for inclusion. Subjects were recruited by an expert gynecologist during regular monitoring of pregnancy, and included in the study only if: 1) normal fetal anatomy was demonstrated at fetal ultrasound assessment at 20 weeks performed by fetal medicine subspecialists, 2) normal fetal growth was demonstrated at ultrasound scan before fetal MRI (estimated fetal weight between 10 and 90 centile at 24–35 weeks, normal fetal Doppler studies), 3) no major maternal diseases or pharmacological treatment influencing fetal metabolism and normal development were present (diabetes mellitus, thyroid dysfunction, chronic hypertension, autoimmune diseases, known thrombophilic statuses, infections, pre-eclampsia), 4) no sign of fetal neurodevelopmental abnormality nor brain parenchymal signal alterations were acknowledged by a specialized neuroradiologist by means of structural MRI investigation, and 5) fetal or maternal movement during the exam did not compromise image acquisition. As a result, one subject was excluded as found to be a tetralogy of Fallot carrier; one subject was excluded due to hydrocephalus and 4 subjects were excluded due to excessive movement during acquisition. A final sample of 16 fetuses (Gestational Week, GW mean 28.4, ±1.8; range 25.4: 32.0) (Mother age mean 31.3, ±6.8; range 19: 42 and see Supplementary Table S1 in Supplementary online material—SOM—for subject-wise details) was thus selected for further preprocessing and analyses. The study protocol was approved by the Ethics Committee of the San Raffaele Hospital and all women provided written informed consent prior to MRI examination.

### Image Acquisition

Fetal MR scanning was performed on a Philips Achieva 1.5 T scanner, using a 16 channel body coil. Mothers were asked not to eat within 2.5 h preceding the MR scanning. All the exams were acquired by a pediatric neuroradiologist and a technician. Structural scans consisted of a T2 Single Shot Turbo Spin Echo scan on the axial, sagittal and coronal planes of the fetus, TR = 8000 ms, TE = 125 ms, voxel size 1.17 × 2.76 × 3 mm, #slices = 25, for a total scanning duration time of 17 s. Functional scans (rs-fMRI) consisted of GE EPI scans TR = 2000 ms, TE = 30 ms, acquisition voxel size 2.81 × 2.86 × 3 mm, # slices = 25, slice gap = 0. Each rs-fMRI scan consisted of 60 volumes lasting 2 s each, for a total scanning time of 2 min per scan. Four to six consecutive rs-fMRI sessions (i.e. 240–360 scans, covering from 8 to 12 min of continuous brain activity at rest) (Van Dijk et al. 2009) were acquired for each subject depending on the patient condition and quality of the scans. Subject-specific details on scans acquisition and inclusion into analysis are provided in Supplementary Table S2 of the Supplementary online material.

### Image Processing

Resting State functional volumes were preprocessed using SPM12 (https://www.fil.ion.ucl.ac.uk/spm/). Resting state fMRI scans were preprocessed using a within-to-between-sessions (WS-to-BS, respectively) approach. Specifically, at the WS level volumes were as follows: 1) manually reoriented and 2) realigned to a session-specific reference image. WS-realigned volumes were then 3) inspected for outliers in terms of movement and signal intensity, at both the scan-to-scan and scan-to-mean levels (https://www.nitrc.org/projects/artifact_detect, Power et al. 2012, 2014). Outlier volumes diagnosed at the WS level were then scrubbed from the dataset while surviving volumes were input into the BS processing level. Images were then 4) masked, in order to broadly remove maternal abdominal tissue for better BS spatial registration, 5) realigned to a common mean functional reference image, 6) inspected and scrubbed for BS outliers. Volumes surviving the BS scrubbing procedure 7) underwent a final step of BS realignment for additional registration, and 8) were stripped using a subject-specific brain segmentation procedure, aimed at removing all the residual non-brain tissue in each scan. Stripped images were finally 9) normalized to a standardized space (i.e. a 28GW fetal template, Gholipour et al. 2017). Spatial normalization of fetal brains with a variable GW range to a common GW reference template space is a conventional approach (Thomason et al. 2013, 2014, 2015, 2017, 2018; Van Den Heuvel et al. 2018; Wheelock et al. 2019; Turk et al. 2019) provided that neuroanatomical consistency is shared across the entire GW range. With respect to our sample (i.e. 25–32 GW) all subjects shared the regional cortical differentiation features (i.e. sulci and fissures) which mature by the end of the fetal period (i.e. 23rd GW) (Kostovic and Jovanov-Milosevic 2006; Kostovic and Vasung 2009; Habas et al. 2012). EPI volumes were finally 10) smoothed with a 4 mm gaussian kernel before entering statistical analysis. Refer to the supplementary online materials (section 1, “Image Processing Details”) for a detailed account of the rationale and specification of each step included in the preprocessing pipeline.

### Statistical Analysis

#### Seed-Based-Connectivity Analysis

Normalized, smoothed volumes were investigated for patterns of subcortico-cortical, seed-to-voxel connectivity using the CONN functional connectivity toolbox (Whitfield-Gabrieli and Nieto Castanon 2012, https://web.conn-toolbox.org/) ver. 18.b. At the first, single subject level, signal was extracted from L and R Thalamus, L and R Hippocampus, L and R Caudate Nucleus, L and R Amygdala, L and R Lentiform Nucleus (Globus Pallidus + Putamen) and L and R Subthalamic Nucleus seeds (defined using the 28 GW fetal brain parcellation provided with the template used for EPI scans normalization) (Gholipour et al. 2017). Principal components of WM and CSF and whole brain signal were estimated using the anatomical component correction (aCompCor) (Behzadi et al. 2007) and regressed out from the first-level analysis. Signal was temporally filtered to retain frequencies in the 0.01–0.08 Hz range, in which intrinsic functional connectivity has been previously reported to occur consistently (Van Dijk et al. 2009) and which has been previously employed to analyze seed-based connectivity in a fetal dataset (Thomason et al. 2013). Bivariate Pearson correlations were then calculated between the timeseries (TS) extracted from each seed region and the TS of every other voxel in the brain. Furthermore, in order to exclude residual non-brain voxels, first-level connectivity maps investigation was spatially constrained using the 28 GW template image as an explicit, inclusive mask. Connectivity maps between each seed and the whole-brain of each subject were then entered into second level, random effects modeling for group comparisons. Maps were tested for both 1) positive, unconstrained increases of connectivity and 2) positive increases of connectivity modulated by the GW at scan. Results were explored using a combined FWE/FDR approach and deemed significant only if reaching significance at both the *P* = 0.05 FWE (voxel level) and *P* = 0.001 FDR levels. Further detail on model specification, estimation and the results thresholding rationale is provided in Section 2 of the supplementary online material (“Statistical Analysis Detail”).

## Results

Table 1 reports the full list of clusters (and their details in terms of localization, significance, and extent) showing a significant connectivity increase with each subcortical seed investigated in the study. Reported clusters localization in terms of cortical districts (e.g. frontal, temporal, cingulate, etc.) and positional brain landmarks (e.g. lateral, medial, inferior, middle, and superior) has been performed on the 28 GW fetal template space (Gholipour et al. 2017), corresponding to the median anatomy of our sample, to which all volumes were normalized for within and between subjects analysis (see above). Group-level biometrical consistency with the 28 GW template was assessed by comparing each subject’s biparietal and fronto-occipital diameters measures (BPD and FOD, respectively) (BPD, millimiters; group mean 60.3 ± 4.2, minimum 52, maximum 68; FOD, millimeters; group mean 79.7 ± 5.2, minimum 69, maximum 89) to normative data provided by Garel (2004) and assessed to fall within ranges of the 28 GW anatomy distribution (BPD range 52–69; FOD range 70–90) (refer to SOM section 4, “Biometrical Consistency” for a report on subject-specific BPD and FOD data). Furthermore, thresholded seed-to-voxel maps of significant subcortico-cortical activation were also normalized to the 25th, 27th, 30th, and 32nd GW template spaces (corresponding to the 1st, 25th, 75th and 100th centiles of the GW distribution of our sample) in order to assess and detail localization consistency across cortical districts and positional landmarks with information on layers neurodevelopmental status across the sample GW range. Details on the normalization procedure and clusters localization consistency are provided in SOM (section 3, “Clusters Localization Consistency”), along with a visual representation of subcortical seeds maturation consistency at the 25, 27, 28, 30 and 32 GW timepoints (SOM, Supplementary Figure S2).

**Table 1:** Clusters of increased connectivity between each seed selected for subcortico-cortical connectivity investigation and the whole-brain revealed by our analyses. Reported results have been investigated using a combined FWE (*P* = 0.05) and FDR (*P* = 0.001) threshold and localized within the Gholipour (2017) 28 GW fetal template coordinates reference system.

Unconstrained connectivity (pFWE = 0.05; pFDR = 0.001)						
Seed	Coordinates				Metrics	Localization
	X	Y	Z	KE	P FWE Corr (peak level)	
L Lentiform	−7.2	4.0	8.8	31	0.001	L Thalamus
(Caudate Nucleus + Globus	1.6	16	6.4	41	0.004	R Caudate Head
Pallidus)	−16.0	11.2	9.6	73	0.002	L Insular Cortex
						
	−8.0	17.6	5.6	11	0.002	L Lentiform
	9.6	21.6	2.4	139	0.001	R Caudate Head
R Lentiform	6.4	3.2	8.0	49	0.001	R Thalamus
	28.0	1.6	3.2	31	0.002	R STG (Primary Auditory)
(Caudate Nucleus + Globus	25.6	−24.0	6.4	29	0.003	R Occipital (Primary Visual)
Pallidus)	19.2	9.6	4.0	29	0.002	R Posterior Insula
	15.2	23.2	1.6	6	0.029	R Anterior Insula
	−1.6	14.4	−0.8	17	0.001	L Ventromedial PreFrontal Cortex
	1.6	31.2	3.2	18	0.014	Dorsal Anterior Cingulate
	−10.4	−1.6	13.6	58	0.007	L Thalamus
	−13.6	11.2	11.2	15	0.007	L Lentiform
	−28.0	1.6	21.6	6	0.017	L Postcentral Gyrus (Somatosensory)
	34.4	6.4	16.0	15	0.016	R Postcentral Gyrus (Somatosensory)
L Caudate Nucleus	21.6	5.6	20.8	20	0.008	R Central Sulcus (Sensorimotor)
	−3.2	−20.8	26.4	15	0.006	L Precuneus
	−11.2	−12.0	22.4	15	0.001	L Superior Parietal (Paracentral)
	19.2	−8.8	19.2	21	0.002	R Angular Gyrus
	−3.2	−7.2	24.0	24	0.001	L Posterior Cingulate Cortex
	−8.0	−0.8	14.4	4	0.024	L Thalamus
R Caudate Nucleus	16.8	15.2	12.0	9	0.021	R Lentiform
	31.2	8.8	6.4	3	0.045	R STG (Primary Auditory)
	−20.0	28.8	18.4	4	0.018	L DLPFC
L Thalamus	−10.4	17.6	12.0	179	0.000	L Lentiform
	8.8	4.8	8.8	2	0.037	R Thalamus
	12.8	10.4	15.2	20	0.005	R Caudate Nucleus
R Thalamus	0.8	−2.4	−1.6	29	0.003	Substantia Nigra
	24.0	−7.2	−4.0	17	0.017	Middle Temporal Gyrus
	−9.6	8.8	−5.6	56	0.000	L Perirhinal (Primary Olfactory)
	−8.8	−16.8	−2.4	112	0.001	L Parahippocampal
	3.2	11.2	−3.2	43	0.003	R Pituitary
L Hippocampus	5.6	−5.6	−3.2	5	0.017	R VTA
	−7.2	−29.6	−4.8	6	0.019	L Posterior Fusiform
	16.8	16.8	−9.6	5	0.038	R Mesial Temporal Pole
	−21.6	16.0	−10.4	27	0.008	L Temporal Pole
	27.2	16.8	−7.2	37	0.006	R Temporal Pole
	−2.4	−1.6	−12.0	68	0.003	L Brainstem (Pons)
	−6.4	−7.2	−6.4	37	0.005	L Cerebellum
	10.4	−16.0	−8.8	24	0.007	R Cerebellum
	−10.4	7.2	−9.6	6	0.012	L Hipp to Temporopolar Cortex
R Hippocampus	6.4	−20.0	3.2	11	0.003	R Cuneus/Calcarine Fissure
	31.2	−3.2	−2.4	104	0.012	R Middle Temporal Gyrus
	−16.8	−18.4	−4.8	9	0.016	L Posterior Fusiform
	28.8	1.6	−7.2	7	0.029	R Middle/Inferior Temporal
	25.6	17.6	−7.2	163	0.001	R Anterior Temporal Pole
L Amygdala	−16.0	9.6	−7.2	328	0.001	L Anterior Temporal Pole
Continued						
R Amygdala	−2,4	−19.2	−12.0	28	0.001	L Cerebellum (to central Lobule)
	19.2	−27.2	−3.2	9	0.018	R Inferior Occipital Gyrus
	12.0	−16.0	−4.0	5	0.028	R Lingual Gyrus
	30.4	−8.8	−4.0	7	0.019	R ITG
Effect of GW increase (i.e. 25–32 GW) on connectivity strength (pFWE = 0.05; pFDR = 0.001)						
SEED	COORDINATES				METRICS	LOCALIZATION
	X	Y	Z	kE	FWE Corr (peak level)	
L Thalamus	23.2	−15.2	−8.8	11	0.007	R ITG

## Discussion

In this study, we used rs-fMRI in a sample of 16 fetuses with normal neurodevelopment to investigate emerging functional connectivity at rest in the third trimester of pregnancy (i.e. 25–32 GW). During this period, rapid axonal migratory waves set the stage for the transition from temporary to permanent patterns of thalamo-cortical afferentation, which is a key ontogenetic step for the emergence and tuning of functional networks in the developing brain (Kostovic and Jovanov-Milosevic 2006). Specifically, a first form of intrinsically driven functional synchronization is reached by the end of the 23rd GW, with thalamic efferents leaving the ventricular zone and accumulating in the subplate (SP) in correspondence of their future cortical targets (Kostovic and Jovanov-Milosevic 2006). Following, thalamo-cortical axons abandon their transient locations in the SP and start migrating to their permanent target layers in the cortical plate (CP) of both primary sensorimotor and associative developing cortices. This process of structural maturation reaches its apex around the 32nd GW and, critically, sets the neurophysiological stage for the establishment of a proper functional cross talk, leading to the emergence of resting state networks in the fetal brain (Thomason et al. 2018). This step in neurophysiological maturation during the early preterm phase (Kostovic and Jovanov-Milosevic 2006) is also paralleled by a major step in behavioral maturation, characterized by the progressive emergence of more complex (DiPietro et al. 2018; Piontelli 2014), integrated (DiPietro et al. 1996) and controlled (Roodenburg et al. 1991; Morokuma et al. 2004) behavioral patterns (see below). We present our findings as a maturational bridge between the neurophysiological and behavioral developmental pillars, moving through pathways of subcortico-cortical functional connectivity directed toward the establishment of solid brain–behavior relationship anchors.

We will now detail our findings on emerging fetal subcortico-cortical functional synchronization in terms of its relevance for the development of some major cognitive abilities, grounded in a foundational “cognitive development blueprint,” through which primary features of cognition are planned to be developed before birth, in order to allow the newborn to properly interact with the extrauterine environment and to survive. We discuss functional maturation of areas and networks based on their relevance for cognition during postnatal life, mirrored to a functional equivalence in the fetus through behaviors observed both during gestation and at birth. To this end, we present the cognitive development blueprint as a theoretical framework, for that no evidence is, at present, available to perform a direct functional-cognitive pairing assessment in the fetus.

### Sensorimotor Processing

First, we highlight the patterns of connectivity found between the DS seeds (i.e. lentiform and caudate nucleus) and the primary auditory and visual cortices, the somatosensory cortices and the primary motor cortex and between the hippocampal seeds and the primary olfactory cortex, the primary visual cortex and the bilateral cerebellum. These fetal connectivity patterns support the view that a substantial functional maturation of areas related to sensorimotor processing is achieved by the third trimester of pregnancy. Our evidence overlaps with cortical structural afferentation reported by Kostovic and colleagues (Kostovic and Jovanov-Milosevic 2006) and demonstrates that structural development is paralleled with functional maturation of primary visual, auditory, and motor cortices already before birth (Schopf et al. 2012; Thomason et al. 2013, 2018; Vazquez-Rodriguez et al. 2019).

No direct functional coupling between the thalamus and sensorimotor cortices emerged. However, thalamic activity was found to be associated to DS activity, which in turn was coupled with sensorimotor areas. Such functional configuration closely resembles results reported in the neonate (Baldoli et al. 2015), with the DS and the thalamus acting together to support voluntary movement control. Such view of a complex maturation of the sensorimotor system is also supported by the patterns of connectivity increase found within the DS (i.e. between the lentiform and the caudate nuclei) and between the thalamus and the substantia nigra in the brainstem. We argue that such overall pattern suggests a consistent functional tuning of areas involved in the direct and indirect pathways, with mesolimbic nuclei acting on the thalamus and the DS for the activation or inhibition of cortical areas deputed to sensorimotor processing (Calabresi et al. 2014). To this end, the lack of significant loci of connectivity for the subthalamic nuclei (STN) could be explained by taking into consideration that functional maturation of the STN has been reported to rely on myelination of long-ranged subcortico-cortical connections (Nambu et al. 2002) which, critically, consistently begins at around the 35th GW and is protracted throughout the postnatal period (Kostovic and Jovanov-Milosevic 2006; de Graaf-Peters and Haddes-Algra 2006). Such consistent patterns of sensorimotor functional maturation are in line with behavioral manifestations of motor maturity observed in both the late fetus and the newborn. Motor behaviors in the fetus emerge as early as the 9 GW and start to manifest as basic arc reflex activity (e.g. startles and stretches). Fetal movements grow in speed, amplitude, and complexity (Roodenburg et al. 1991) throughout the first and second trimester, while decrease during the third trimester (Kurjak et al. 2005).

**Figure 1:**
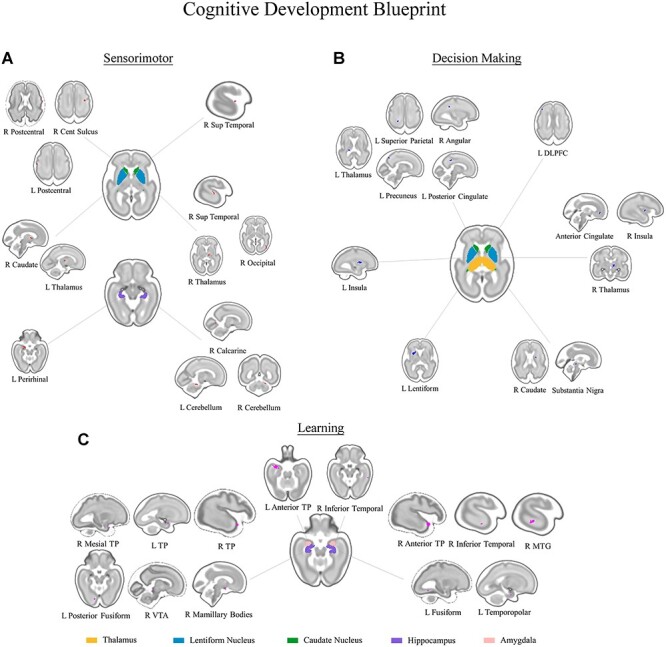
Functional synchronization patterns underlying the “Cognitive Development Blueprint,” a set of “cognitive tools” developed during gestation in order to allow the incoming newborn a successful interaction with the extrauterine environment (i.e. allowing for survival). (*a*) “Sensorimotor” (upper left) functionality synchronization, involving the maturation of a complex network of cortical, subcortical, and mesolimbic processing loci supporting environmental perception and voluntary control of movement. (*b*) “Decision Making” (upper right) functionality synchronization, involving the maturation of networks (i.e. salience network and DMN) underlying the management of attentional resources, supporting selection, and proper execution of survival-oriented behaviors. (*c*) “Learning” (bottom) functionality synchronization involving the maturation of the medial temporal lobe memory system, as well as of temporal structures supporting the ability to represent, store and retrieve gathered information at different levels of complexity.

The reduction of fetal movements during late gestation has been directly paralleled with a major step in the maturation of the central nervous system and, particularly, of sensorimotor integration (Kurjak et al. 2005; DiPietro et al. 2018; Piontelli 2014). Accordingly, multiple direct behavioral manifestations of such functional maturation are manifest immediately after birth in terms of the available repertoire, smoothness, and coordination of movements (Als 1982). The ontogenetic meaning of this fetal functional sensorimotor subcortico-cortical configuration relies in the capability of newborn: *i) to process peripheral sensory information gathered through senses* and *ii) to properly execute behavioral responses* (see Fig. 1). The relevance of such capabilities is outstanding for survival in that sensory perception allows for the creation of a representation of the world, while motor execution to interact with it; in other words, no form of cognition would be possible in the absence of such functional configuration.

### Decision Making

Sensorimotor processing is not sufficient for adaptation of the newborn to the outer world and for the emergence of higher-order cognition. Our results also highlighted a pattern of significant connectivity between the DS, and particularly the putamen, and both the Iinsular (IC) and anterior cingulate (ACC) cortices. These areas are known as the two major cortical seeds of the salience network (SN), which underlies the ability to orient attentional resources toward stimuli which are relevant for the selection and execution of goal-directed behaviors (Seeley et al. 2007). To this end, we would like to draw the reader’s attention to the so called “breast crawling” phenomenon, a spontaneous behavior occurring within few minutes after birth, by which the newborn instinctively uses breast odor information to orient and crawls up the mother’s abdomen to reach the breast and feed (Varendi and Porter 2001). Our results may reveal the functional underpinnings for such behavior to occur: the SN could be at play to drive attention toward the salient peripheral olfactory information; this, in turn, would be relayed through the thalamus to sensory cortices for explicit processing and finally used to control for a motor response by means of striatal modulation (Peters et al. 2016). While exhaustive, this account lacks in the explanation of how stimuli without an intrinsic relevance are processed for salience attribution. To this end, the pattern of activation found for dopaminergic nuclei of the brainstem points toward a functional maturation of reward-based signaling pathways. In addition to their sensorimotor involvement, these areas respond to stimulus novelty and regulate approach and avoidance responses through perceived hedonic valence (Bunzeck and Dünzel 2006; Roitman et al. 2008). Accordingly, newborn-world interactions associated to a sensation of reward could be assigned with a positive valence, marked as salient and associated to approaching behaviors. Valence would, in turn, be used to increase adaptation efficiency, by biasing world exploration toward salient configuration of stimuli to progressively fine-tune selection, execution and monitoring of behavioral responses. Of notice, salience attribution has been revealed to occur during the last trimester of pregnancy, with fetuses showing selective behavioral modifications (i.e. strengthening or extinction of a behavior) in response to repeated exposure to stimuli from the extrauterine environment (James 2010 and see the next paragraph). Our results also highlighted a broad pattern of connectivity between the DS and both the posterior (i.e. posterior cingulate cortex, precuneus, angular gyrus and superior parietal cortex) and anterior (i.e. ventromedial prefrontal cortex—vm/DLPFC) seeds of the default mode network (DMN) (Raichle et al. 2001). Reported to be consistently associated to the retrieval of autobiographical memories (Philippi et al. 2014), we speculate that functional maturation of this network at birth could underlie the ability for the newborn to switch from external to internal cognition. With respect to salience/valence attribution this ability would, in turn, allow to perform an internal review of experiences, in order to assign and modulate salience and valence without necessarily experiencing it. More specifically, as a result of the reviewing process, a decision will lead toward direct salience attribution or toward the necessity to re-experience a certain aspect of the world in order to reach a cognitive verdict on its relevance for survival. The role of a prototypical DMN configuration at this stage in development may cover a crucial cognitive foundation, i.e. the ability to build autobiographical information on experience, functionally pretrained during gestation and realized through experience during postnatal life. In line with this interpretation previous studies have reported that a prototypical configuration of the DMN is already traceable in the fetal brain, while its functional transition into an adult-like form requires approximately one year of postnatal life to complete (Alcauter et al. 2014).

We thus conclude that functional maturation of connectivity between the DS and the thalamus, between the DS and SN and DMN cortical processing sites and within the DS itself supports the ability for the newborn to *iii) integrate sensory information into cognition in order to create salient representations of the world* (see Fig. 1). These representations are, in turn, needed to improve the efficiency of newborn-world interactions and, thus, to maximize survival.

### Learning

In the previous paragraph, we highlighted the relevance of information retrieval for salience attribution. However, a functional maturation of learning-related circuitry was also observed through a significant pattern of positive connectivity between the hippocampal seeds, the mamillary bodies (Vann and Nelson 2015) and several areas of the bilateral temporal lobe (TL), extending to both medial and lateral aspects of the middle and inferior temporal cortices and ranging from the fusiform gyrus (FG) to the polar regions. Temporal regions are known to play a crucial role for learning and retrieval to occur. The inferior temporal gyrus (ITG), for instance, acts as the processing terminal of the ventral visual pathway, allowing for the recognition of complex representations of stimuli (e.g. objects) based on their spatial and color features (Amedi et al. 2001). The FG plays a crucial role for fine-grained, within-category features recognition and, interestingly, shows a functional specialization for the processing of faces (Peelen and Downing 2005; Grill-Spector et al. 2004). Finally, the polar temporal regions underlie processing of amodal representations of knowledge and are thus considered to be major cortical sites for semantics (Lambon Ralph et al. 2008). Together with adjacent enthorinal, perirhinal and parahippocampal cortices, the hippocampus acts as a hub through which information processed and stored across different cortical sites is integrated and stored into long-term memories (Squire and Zola-Morgan 1991). To this end, functional maturation of temporal cortices may subserve the ontogenetic priority to provide the newborn with the cognitive ability to recognize coherent perceptual clusters of information, allowing for the creation of meaningful representations of the world. Such representations would then be stored at different temporal cortical sites and then integrated through hippocampal activity for storage into long-term memory traces. The relevance of such cognitive ability for survival is outstanding per se and especially when considering its functional interaction with salience attribution: the ability to process, represent, and retrieve information at different levels of complexity and abstraction (i.e. ranging from within-category specific features to amodal representations) is, in fact, pivotal to tune salience attribution in order to adapt to specific goals and drive self-motivation. For instance, during the initial phase of maternal face exploration, a newborn will learn to detect specific manifestations of both positive (e.g. happy faces) and negative (e.g. threatening faces) emotions (Farroni et al. 2007). Based on this experience, he would then learn to generalize salient features allowing him to recognize the emotion independently from a specific face. Complementary, over time, he would also learn to detect and use specific features, allowing him to recognize subtle nuances in the expression of the same emotion between faces of specific individuals (Turati and Simion 2002). Such an ability can be argued to be critical in the first phases of life, being the interaction with conspecifics a defining feature of survival for newborns in our specie (Rosenberg 1992).

The pattern of association found between the hippocampi and dopaminergic nuclei of the ventral tegmental area (VTA) in the brainstem suggests for a functional maturation of reward-based modulation of learning. Taken together with its effect on striatal activity, dopamine release could thus be at play acting both as a salience marker and as a cue to establish a long-term trace of relevant information. Functional modulation of hippocampal activity has been observed, for instance, in response to salient stimuli retrieval (Ungless 2004) and to consolidation of goal-directed behaviors (Luo et al. 2011). Complementary, dopaminergic dysregulation has been observed in association to the emergence of psychotic symptoms which are, critically, characterized by aberrant salience attribution (Winton-Brown et al., 2014).

The pattern of connectivity found between the amygdala and the occipital and temporal (ITG and polar) cortices further suggests for the maturation of another critical source of salience-modulated learning. Amygdala activation has in fact been recently reported to be involved in processing the relevance of stimuli (Cunningham and Brosch 2012), with its activation being critically modulated by salience and regardless of the emotional valence (Santos et al., 2011). Nevertheless, we suggest maturation of the amygdala-temporal synchronization at birth to be likely associated to amygdala specialization in detecting threatening stimuli (Anderson and Phelps 2001). This structure could thus mediate the ability to emotionally strengthen the detection and attribution of aversive salience, as a preferential way for the consolidation of avoidance behaviors toward potentially noxious stimuli. Accordingly, activation of the amygdala, along with temporal areas, has been reported in response to low spatial frequency stimuli, such as fearful faces (Johnson 2005), which elicit a preferential response in the newborn. As a final notice, we would also like to highlight that the thalamus also showed a significant increase as a function of increasing GW and that this connectivity targeted the inferior temporal region. This could suggest that substantial functional maturation of the memory system would start following the maturation of functional networks underlying sensorimotor processing and decision making. Such a developmental trajectory would make sense in ontogenetic terms, with memory consolidation functionality development occurring once the structural and functional infrastructure regulating information selection and behavioral control have been established and, thus, when adaptive interactions with the world are ready to be formed and stored for survival. In line with these results, salience-driven learning capabilities have been demonstrated to consistently emerge in the fetus at around the 28–32 GW range (Krueger et al. 2004). Exposure learning paradigms have, for instance, revealed that the effects (i.e. selective behavioral responses) of exposure to maternal voice (DeCasper and Fifer 1986; Moon and Fifer 2000) and music (James et al. 2002) during the last weeks of gestation are protracted in the postnatal period, thus revealing that a form of stimulus salience attribution and consequent learning can occur in the fetal brain. Habituation learning paradigms have revealed that habituation induced during pregnancy occurs faster (i.e. lower number of repetitions necessary for a behavioral change to occur) as gestational age increases (Morokuma et al. 2008) and that fetuses showing faster habituation learning rates exhibit higher motor maturity during gestation (Morokuma et al. 2004), more efficient environmental adaptation at birth (in terms of a wider repertoire of behavioral states) and higher efficiency in attention orientation shortly after birth (i.e. 2 and 10 days) (Madison et al. 1986). The occurrence of habituation learning already during fetal life reveals the emergence of a progressively more environment-oriented brain–behavior relationship, by which external stimuli are evaluated, classified and (eventually) functionally integrated into cognition. In line with this interpretation, our results revealed functional maturation of paralimbic cortices (i.e. temporopolar, ventromedial prefrontal, parahippocampal, and cingulate) which, as stated by Mesulam and Mufson (1982) “provide a neural apparatus for mediating between the external environment and the internal milieu.” To this end we further stress our previously discussed finding on the development of protoypical DMN functionality, and particularly of the medial TL subsystem (Buckner et al. 2008) by suggesting that fetal DMN maturation could represent a critical asset for the formation of autobiographical memory during childhood and in the adult. During the third trimester of pregnancy the fetus: 1) lives in an intrauterine environment which is not fully deprived of the light and sound (Magoon and Robb 1981), 2) is able to process tactile, vestibular, taste, olfactory, auditory, and visual sensations at the cortical level (Kostovic and Jovanov-Milosevic 2006; Kostovic and Judas 2010; Kostovic et al. 2019) and 3) reacts to environmental stimulations (Piontelli 2014; Morokuma et al. 2004). We move from the hypothesis of Margulies et al. (2016) by which DMN development is driven by a principal gradient, targeted at functionally binding perception and action to cognition. Under this assumption, the DMN is equidistant from somatosensory, visual, and auditory systems and is not influenced by external stimulation (Buckner et al. 2019). Instead, it acts as a functional hub for the integration of information coming from perception and action into prototypical forms of memory, which will converge later on in ontogeny in an autobiographical cognition of continuity between past and future memories of the child and the adult. Accordingly, long-lasting rudimental traces of fetal memory have been shown in the auditory cortex even before the brain has reached full-term maturation (Granier-Deferre et al. 2011) and we believe that subcortico-cortical connections are required for the functional linkage between internal and external components of development (Brusseau 2008).

We conclude by presenting learning as the final component of the “cognitive development blueprint.” With respect to survival-oriented cognition we suggest the maturation of this functional system to underlie three main abilities, i.e. *iv) to store salient information, v) to recover it in order to strengthen or remodulate salience, vi) to fasten and enhance the salience attribution through emotional and reward related processing of stimuli* (see Fig. 1).

### Pathological Impairment Gradient of the Cognitive Development Blueprint

Early life cognitive impairments emerge as a consequence of deviations from normal brain development. In some cases, such as major brain malformations, these abnormalities entail macroscopic alterations of cerebral structures while in others alterations are found at the microstructural level and reflected in altered patterns of functionality. From the behavioral point of view, the former usually manifest immediately or soon after birth as overt focal cognitive impairments, while the latter are more likely to emerge later during development, with a variable impact on cognition in terms of severity and specificity of affected cognitive abilities. For instance, developmental dyslexia entails a specific impairment in reading ability despite normal intelligence and sensory abilities (Demonet et al. 2004), with its functional underpinnings manifesting as hypoactivation of reading-related cortices (e.g. fusiform and extrastriate) (Maisog et al. 2008) which, in turn, affect functionality of whole reading-related systems (e.g. the posterior system) (Pugh et al. 2000). Along similar lines, attention deficit hyperactivity disorder (ADHD) involves a substantial impairment of the attentional regulatory systems, in terms of both salience detection and cognitive control abilities. Given its pivotal role in cognition (Simon 1986), early impairments of attention are frequently found to affect functionality of multiple domains and often manifest in the form of diverse developmental learning disabilities (DuPaul and Volpe 2009). Critically, this variability in behavioral manifestations is paralleled by patterns of altered functionality in terms of whole networks, rather than specific areas. More in detail, the ADHD brain is characterized by a marked pattern of altered functional synchronization both: 1) within networks underlying salience detection and executive control and 2) between these networks and the regulatory activity of DMN (Castellanos and Proal 2012). Taking one step further, autistic spectrum disorders (ASD) could be considered amongst the most faceted clinical conditions in terms of manifest cognitive impairments. Subjects with ASD show an outstanding variability in terms of behavioral manifestations, involving impairments in terms of sensorimotor functionality (Mosconi and Sweeney 2015), memory (Boucher et al. 2012), attention (Tye et al. 2013), and social cognition (Senju 2012). Similar to ADHD, the ASD brain is also characterized by a system-level impairment, involving anomalous synchronization of networks related to cognitive control. More than ADHD, this anomaly also features a prominent within and between networks underconnectivity (Just et al. 2012; Kana et al. 2014; Hughes 2007). In our consideration, the above-cited examples suggest the existence of two gradients, proceeding in parallel and accounting for the impact of abnormal neurodevelopment on survival: 1) a functional gradient ranging from area-specific to network and system level dysfunctionality and 2) a cognitive gradient, involving the dysregulation of specific to multiple aspects of cognition. Critically, it appears that the more systematic and cognitively faceted the pathological condition (i.e. ASD), the higher the probability of disruption to nuclear cognitive skills.

Interestingly, major disruptions of cognition have been also observed as consequence of preterm deliveries, with severity of the impairment decreasing as an inverse function of gestational age at birth (Rose et al. 2005). Of notice, ASD frequently occurs as a consequence of extremely premature deliveries (i.e. < 25 GW). It is thus not surprising to observe that ASD dysfunctionality targets the nuclear features of the “cognitive development blueprint,” for that such an abrupt interruption of the unraveling blueprint is expected to undermine the very foundations of cognition. In line with this interpretation ASD (along with severe preterm birth) has been historically associated to epilepsy of the TL, a condition which is highly disruptive for cognition (Levisohn 2007). Interestingly, the TL has been recently found to be, together with the insula, a main fetal cortical site for the propagation of instructive spontaneous activity transients, playing a critical role for normal development of the fetal functional connectome (Thomason 2018; Arichi et al. 2017).

We conclude by suggesting the “cognitive development blueprint” to be a promising framework of research for our understanding of the complex dynamics linking structural and functional development to the emergence of normal and deviant behavioral outcomes.

## Conclusion

Our results revealed a consistent functional maturation of subcortico-cortical networks in the fetal brain, underlying the development of the cognitive infrastructure which will allow the newborn to survive in the extrauterine environment. Specifically, we suggest the newborn to possess the necessary cognitive infrastructure to: 1) gather and process sensorimotor information, in order to perceive the world and to interact with it; 2) to assign salience, in order to create representations of the world which are relevant to selection and execution of adaptive behaviors; and 3) to store, retrieve, and evaluate the outcomes of interactions with the world, in order to gradually improve both the selection and execution of adaptive behaviors. This functional maturation may well be a natural compensative phenomenon to maximize survival when risk of prematurity increases: premature birth occurring over the 32nd week of gestation is, in fact, more frequently observed and presents higher chance of both survival and intact neurodevelopment. Under this light, the observed functional maturation could thus reflect the effect of evolutionary pressure for the selection of nuclear cognitive abilities, maximizing the probability of survival of the newborn in the outer world. We thus present our results as revelatory of a “cognitive development blueprint.”

In a recently published, outstanding work Turk and collaborators (2019) have shown the functional connectome of the fetal brain to possess a consistent degree of maturation, in terms of both networks' synchronization and strength. We believe cortico-cortical networks highlighted in their findings on a considerably larger sample (*n* = 105) to strongly support the maturation of cognitive abilities we presented as nuclear features of the “cognitive development blueprint.” The limited size of our sample could have contributed to the over- or underestimation of the observed patterns of connectivity. Therefore we suggest that a direct investigation of the fetal functional subcorticocortical connectome with a larger sample size would be of great interest to our understanding of fetal cognitive ability development. Extensively, we suggest that further characterization of fetal neurodevelopment should pass through four major steps involving 1) specification of the precise timing of emergence of major functional maturation milestones (including the cognitive development blueprint components), which will be critical to discern normal from deviant functional development throughout gestation; 2) the integration of subcortico-cortical and cortico-cortical structural and functional connectomes; 3) the use of follow-up designs, aimed at tracing developmental trajectories from the fetal to the neonatal stage and, possibly, throughout the lifespan; and 4) direct investigations of how structural and functional features of the connectomes relate to behaviorally measured cognitive profiles of normal and pathological fetal and neonatal populations. Further, the assessment of fetal behaviors (both in terms of spontaneous and elicited activity) has been consistently reported as a unique window on central nervous system maturation, with behavioral profiles observed during gestation predicting both the emergence of pathology (James 2010; Morokuma et al. 2004) and interindividual behavioral differences in the newborn and during childhood (DiPietro et al. 1996, 2018; Piontelli 2014). Nevertheless, as stated by Morokuma and collaborators in their 2004 work on fetal habituation “[...] it is still impossible to evaluate higher CNS function of the fetus, including that of the cerebral hemispheres, in utero.” We firmly believe that functional magnetic resonance of the fetal brain will prove to be the means to overcoming such a hurdle. Direct observation of functional maturation, together with investigation of its behavioral manifestations, will provide the necessary information to establish ontogenetic neurodevelopmental trajectories. These, in turn, will allow for a specific prediction of cognitive and behavioral neonatal outcomes during gestation and will prove critical to tailor neonatal care on a subject-specific base. Contextually we believe that a big contribution to our understanding would also emerge by comparison of the human and other species structural, functional, and behavioral maturation. Unearthing the link between varying degrees of behavioral complexity and their structural and functional underpinnings will, in fact, likely provide a great contribution in disclosing the very nuclear phylogenetic features of our brain.

## Supplementary Material

SOM_Canini_Final_CCC_tgaa008

## References

[ref1] Alcauter S , Lin W, Smith JK, Short SJ, Goldman BD, Reznick JS, Gilmore JH, Gao W. 2014. Development of thalamocortical connectivity during infancy and its cognitive correlations. Journal of Neuroscience. 34:9067–9075. 24990927 10.1523/JNEUROSCI.0796-14.2014 PMC4078084

[ref2] Als H. 1982. Toward a synactive theory of development: promise for the assessment and support of infant individuality. Infant Mental Health Journal. 3:229–243.

[ref3] Amedi A , Malach R, Hendler T, Peled S, Zohary E. 2001. Visuo-haptic object-related activation in the ventral visual pathway. Nature Neuroscience. 4:324. 11224551 10.1038/85201

[ref4] Anderson AK , Phelps EA. 2001. Lesions of the human amygdala impair enhanced perception of emotionally salient events. Nature. 411:305. 11357132 10.1038/35077083

[ref5] Arichi T , Whitehead K, Barone G, Pressler R, Padormo F, Edwards AD, Fabrizi L. 2017. Localization of spontaneous bursting neuronal activity in the preterm human brain with simultaneous EEG-fMRI. eLife. 6:e27814. 28893378 10.7554/eLife.27814 PMC5595428

[ref6] Baldoli C , Scola E, Della Rosa PA, Pontesilli S, Longaretti R, Poloniato A, Scotti R, Blasi V, Cirillo S, Iadanza A. 2015. Maturation of preterm newborn brains: a fMRI–DTI study of auditory processing of linguistic stimuli and white matter development. Brain Structure and Function. 220:3733–3751. 25244942 10.1007/s00429-014-0887-5

[ref7] Behzadi Y , Restom K, Liau J, Liu TT. 2007. A component based noise correction method (CompCor) for BOLD and perfusion based fMRI. NeuroImage. 37:90–101. 17560126 10.1016/j.neuroimage.2007.04.042 PMC2214855

[ref8] Boucher J , Mayes A, Bigham S. 2012. Memory in autistic spectrum disorder. Psychological Bulletin. 138:458. 22409507 10.1037/a0026869

[ref9] Brusseau R. 2008. Developmental perspectives: is the fetus conscious? International Anesthesiology Clinics. 46:11–23. 10.1097/AIA.0b013e318181a88e 18617815

[ref10] Buckner RL , Andrews-Hanna JR, Schacter DL. 2008. The brain's default network: anatomy, function, and relevance to disease. Annals of the New York Academy of Sciences. 1124:1–38. 18400922 10.1196/annals.1440.011

[ref11] Buckner RL , DiNicola LM. 2019. The brain’s default network: updated anatomy, physiology and evolving insights. Nature Reviews Neuroscience. 20:593–608. 31492945 10.1038/s41583-019-0212-7

[ref12] Bunzeck N , Düzel E. 2006. Absolute coding of stimulus novelty in the human substantia nigra/VTA. Neuron. 51:369–379. 16880131 10.1016/j.neuron.2006.06.021

[ref13] Calabresi P , Picconi B, Tozzi A, Ghiglieri V, Di Filippo M. 2014. Direct and indirect pathways of basal ganglia: a critical reappraisal. Nature Neuroscience. 17:1022. 25065439 10.1038/nn.3743

[ref14] Cao M , Wang J-H, Dai Z-J, Cao X-Y, Jiang L-L, Fan F-M, Song X-W, Xia M-R, Shu N, Dong Q. 2014. Topological organization of the human brain functional connectome across the lifespan. Developmental Cognitive Neuroscience. 7:76–93. 24333927 10.1016/j.dcn.2013.11.004 PMC6987957

[ref15] Castellanos FX , Proal E. 2012. Large-scale brain systems in ADHD: beyond the prefrontal–striatal model. Trends in Cognitive Sciences. 16:17–26. 22169776 10.1016/j.tics.2011.11.007 PMC3272832

[ref16] Conte G , Parazzini C, Falanga G, Cesaretti C, Izzo G, Rustico M, Righini A. 2016. Diagnostic value of prenatal MR imaging in the detection of brain malformations in fetuses before the 26th week of gestational age. American Journal of Neuroradiology. 37:946–951. 26721771 10.3174/ajnr.A4639 PMC7960315

[ref17] Cunningham WA , Brosch T. 2012. Motivational salience: amygdala tuning from traits, needs, values, and goals. Current Directions in Psychological Science. 21:54–59.

[ref18] de Graaf-Peters VB , Hadders-Algra M. 2006. Ontogeny of the human central nervous system: what is happening when? Early Human Development. 82:257–266. 16360292 10.1016/j.earlhumdev.2005.10.013

[ref19] DeCasper AJ , Spence MJ. 1986. Prenatal maternal speech influences newborns' perception of speech sounds. Infant Behavior & Development. 9:133–150.

[ref20] Démonet J-F , Taylor MJ, Chaix Y. 2004. Developmental dyslexia. The Lancet. 363:1451–1460. 10.1016/S0140-6736(04)16106-0 15121410

[ref21] DiPietro JA , Hodgson DM, Costigan KA, Johnson TR. 1996. Fetal antecedents of infant temperament. Child Development. 67:2568–2583. 9022257

[ref22] Dipietro JA , Voegtline KM, Pater HA, Costigan KA. 2018. Predicting child temperament and behavior from the fetus. Development and Psychopathology. 30:855–870. 30068417 10.1017/S0954579418000482 PMC9590372

[ref23] DuPaul GJ , Volpe RJ. 2009. ADHD and learning disabilities: research findings and clinical implications. Current Attention Disorders Reports. 1:152.

[ref24] Farroni T , Menon E, Rigato S, Johnson MH. 2007. The perception of facial expressions in newborns. The European Journal of Developmental Psychology. 4:2–13. 20228970 10.1080/17405620601046832 PMC2836746

[ref25] Garel C . 2004. MRI of the Fetal Brain. Berlin: Springer.

[ref26] Gholipour A , Rollins CK, Velasco-Annis C, Ouaalam A, Akhondi-Asl A, Afacan O, Ortinau CM, Clancy S, Limperopoulos C, Yang E. 2017. A normative spatiotemporal MRI atlas of the fetal brain for automatic segmentation and analysis of early brain growth. Scientific Reports. 7:476. 28352082 10.1038/s41598-017-00525-w PMC5428658

[ref27] Granier-Deferre C , Bassereau S, Ribeiro A, Jacquet A-Y, DeCasper AJ. 2011. A melodic contour repeatedly experienced by human near-term fetuses elicits a profound cardiac reaction one month after birth. PLoS One. 6: e17304. 21383836 10.1371/journal.pone.0017304 PMC3044162

[ref28] Grill-Spector K , Knouf N, Kanwisher N. 2004. The fusiform face area subserves face perception, not generic within-category identification. Nature Neuroscience. 7:555. 15077112 10.1038/nn1224

[ref29] Habas PA , Scott JA, Roosta A, Rajagopalan V, Kim K, Rousseau F, Barkovich AJ, Glenn OA, Studholme C. 2012. Early folding patterns and asymmetries of the normal human brain detected from in utero MRI. Cerebral Cortex. 22:13–25. 21571694 10.1093/cercor/bhr053 PMC3236791

[ref30] Hughes JR . 2007. Autism: the first firm finding= underconnectivity? Epilepsy & Behavior. 11:20–24. 17531541 10.1016/j.yebeh.2007.03.010

[ref31] Jakab A , Schwartz E, Kasprian G, Gruber GM, Prayer D, Schöpf V, Langs G. 2014. Fetal functional imaging portrays heterogeneous development of emerging human brain networks. Frontiers in Human Neuroscience. 8:852. 25374531 10.3389/fnhum.2014.00852 PMC4205819

[ref32] James D , Spencer C, Stepsis B. 2002. Fetal learning: a prospective randomized controlled study. Ultrasound in Obstetrics and Gynecology. 20:431–438. 12423478 10.1046/j.1469-0705.2002.00845.x

[ref33] James DK . 2010. Fetal learning: a critical review. Infant and Child Development. 19:45–54.

[ref34] Johnson MH . 2005. Subcortical face processing. Nature Reviews Neuroscience. 6:766. 16276354 10.1038/nrn1766

[ref35] Just MA , Keller TA, Malave VL, Kana RK, Varma S. 2012. Autism as a neural systems disorder: a theory of frontal-posterior underconnectivity. Neuroscience and Biobehavioral Reviews. 36:1292–1313. 22353426 10.1016/j.neubiorev.2012.02.007 PMC3341852

[ref36] Kana RK , Uddin LQ, Kenet T, Chugani D, Müller R-A. 2014. Brain connectivity in autism. Frontiers in Human Neuroscience. 8:349. 24917800 10.3389/fnhum.2014.00349 PMC4041005

[ref37] Kostović I , Jovanov-Milošević N. 2006. The development of cerebral connections during the first 20–45 weeks’ gestation, Seminars in Fetal and Neonatal Medicine. Amsterdam: Elsevier, pp. 415–422. 10.1016/j.siny.2006.07.001 16962836

[ref38] Kostović I , Judaš M. 2010. The development of the subplate and thalamocortical connections in the human foetal brain. Acta Paediatrica. 99:1119–1127. 20367617 10.1111/j.1651-2227.2010.01811.x

[ref39] Kostović I , Sedmak G, Judaš M. 2019. Neural histology and neurogenesis of the human fetal and infant brain. NeuroImage. 188:743–773. 30594683 10.1016/j.neuroimage.2018.12.043

[ref40] Kostovic I , Vasung L. 2009. Insights from in vitro fetal magnetic resonance imaging of cerebral development, Seminars in perinatology. Amsterdam: Elsevier, p. 220–233. 10.1053/j.semperi.2009.04.003 19631083

[ref41] Krueger C , Holditch-Davis D, Quint S, DeCasper A. 2004. Recurring auditory experience in the 28-to 34-week-old fetus. Infant Behavior & Development. 27:537–543.

[ref42] Kurjak A , Carrera JM, Medic M, Azumendi G, Andonotopo W, Stanojevic M. 2005. The antenatal development of fetal behavioral patterns assessed by four-dimensional sonography. The Journal of Maternal-Fetal & Neonatal Medicine. 17:401–416. 16009643 10.1080/14767050400029657

[ref43] Lambon Ralph MA , Pobric G, Jefferies E. 2008. Conceptual knowledge is underpinned by the temporal pole bilaterally: convergent evidence from rTMS. Cerebral Cortex. 19:832–838. 18678765 10.1093/cercor/bhn131

[ref44] Levisohn PM . 2007. The autism-epilepsy connection. Epilepsia. 48:33–35. 10.1111/j.1528-1167.2007.01399.x 18047599

[ref45] Luo AH , Tahsili-Fahadan P, Wise RA, Lupica CR, Aston-Jones G. 2011. Linking context with reward: a functional circuit from hippocampal CA3 to ventral tegmental area. Science. 333:353–357. 21764750 10.1126/science.1204622 PMC3150711

[ref46] Madison LS , Madison JK, Adubato SA. 1986. Infant behavior and development in relation to fetal movement and habituation. Child Development. 57:1475–1482. 3802972

[ref47] Magoon EH , Robb RM. 1981. Development of myelin in human optic nerve and tract: a light and electron microscopic study. Archives of Ophthalmology. 99:655–659. 7224936 10.1001/archopht.1981.03930010655011

[ref48] Maisog JM , Einbinder ER, Flowers DL, Turkeltaub PE, Eden GF. 2008. A meta-analysis of functional neuroimaging studies of dyslexia. Annals of the New York Academy of Sciences. 1145:237–259. 19076401 10.1196/annals.1416.024

[ref49] Margulies DS , Ghosh SS, Goulas A, Falkiewicz M, Huntenburg JM, Langs G, Bezgin G, Eickhoff SB, Castellanos FX, Petrides M. 2016. Situating the default-mode network along a principal gradient of macroscale cortical organization. Proceedings of the National Academy of Sciences. 113:12574–12579. 10.1073/pnas.1608282113 PMC5098630 27791099

[ref50] Mesulam MM , Mufson EJ. 1982. Insula of the old world monkey. Architectonics in the insulo-orbito-temporal component of the paralimbic brain. The Journal of Comparative Neurology. 212:1–22. 7174905 10.1002/cne.902120102

[ref51] Moon CM , Fifer WP. 2000. Evidence of transnatal auditory learning. Journal of Perinatology. 20:S37–S44. 11190699 10.1038/sj.jp.7200448

[ref52] Morokuma S , Fukushima K, Kawai N, Tomonaga M, Satoh S, Nakano H. 2004. Fetal habituation correlates with functional brain development. Behavioural Brain Research. 153:459–463. 15265643 10.1016/j.bbr.2004.01.002

[ref53] Morokuma S , Doria V, Ierullo A, Kinukawa N, Fukushima K, Nakano H, Arulkumaran S, Papageorghiou AT. 2008. Developmental change in fetal response to repeated low-intensity sound. Developmental Science. 11:47–52. 18171366 10.1111/j.1467-7687.2007.00646.x

[ref54] Mosconi MW , Sweeney JA. 2015. Sensorimotor dysfunctions as primary features of autism spectrum disorders. Science China. Life Sciences. 58:1016–1023. 26335740 10.1007/s11427-015-4894-4 PMC5304941

[ref55] Nambu A , Tokuno H, Takada M. 2002. Functional significance of the cortico–subthalamo–pallidal ‘hyperdirect’pathway. Neuroscience Research. 43:111–117. 12067746 10.1016/s0168-0102(02)00027-5

[ref56] Peelen MV , Downing PE. 2005. Selectivity for the human body in the fusiform gyrus. Journal of Neurophysiology. 93:603–608. 15295012 10.1152/jn.00513.2004

[ref57] Peters SK , Dunlop K, Downar J. 2016. Cortico-striatal-thalamic loop circuits of the salience network: a central pathway in psychiatric disease and treatment. Frontiers in Systems Neuroscience. 10:104. 28082874 10.3389/fnsys.2016.00104 PMC5187454

[ref58] Philippi CL , Tranel D, Duff M, Rudrauf D. 2014. Damage to the default mode network disrupts autobiographical memory retrieval. Social Cognitive and Affective Neuroscience. 10:318–326. 24795444 10.1093/scan/nsu070 PMC4350487

[ref59] Piontelli A . 2014. Development of Normal Fetal Movements. Berlin: Springer.

[ref60] Power JD , Barnes KA, Snyder AZ, Schlaggar BL, Petersen SE. 2012. Spurious but systematic correlations in functional connectivity MRI networks arise from subject motion. NeuroImage. 59:2142–2154. 22019881 10.1016/j.neuroimage.2011.10.018 PMC3254728

[ref61] Power JD , Mitra A, Laumann TO, Snyder AZ, Schlaggar BL, Petersen SE. 2014. Methods to detect, characterize, and remove motion artifact in resting state fMRI. NeuroImage. 84:320–341. 23994314 10.1016/j.neuroimage.2013.08.048 PMC3849338

[ref62] Pugh KR , Mencl WE, Jenner AR, Katz L, Frost SJ, Lee JR, Shaywitz SE, Shaywitz BA. 2000. Functional neuroimaging studies of reading and reading disability (developmental dyslexia). Mental Retardation and Developmental Disabilities Research Reviews. 6:207–213. 10982498 10.1002/1098-2779(2000)6:3<207::AID-MRDD8>3.0.CO;2-P

[ref63] Raichle ME , MacLeod AM, Snyder AZ, Powers WJ, Gusnard DA, Shulman GL. 2001. A default mode of brain function. Proceedings of the National Academy of Sciences. 98:676–682. 10.1073/pnas.98.2.676 PMC14647 11209064

[ref64] Raznahan A , Shaw PW, Lerch JP, Clasen LS, Greenstein D, Berman R, Pipitone J, Chakravarty MM, Giedd JN. 2014. Longitudinal four-dimensional mapping of subcortical anatomy in human development. Proceedings of the National Academy of Sciences. 111:1592–1597. 10.1073/pnas.1316911111 PMC3910572 24474784

[ref65] Roitman MF , Wheeler RA, Wightman RM, Carelli RM. 2008. Real-time chemical responses in the nucleus accumbens differentiate rewarding and aversive stimuli. Nature Neuroscience. 11:1376. 18978779 10.1038/nn.2219 PMC3171188

[ref66] Roodenburg P , Wladimiroff J, Van Es A, Prechtl H. 1991. Classification and quantitative aspects of fetal movements during the second half of normal pregnancy. Early Human Development. 25:19–35. 2055173 10.1016/0378-3782(91)90203-f

[ref67] Rose SA , Feldman JF, Jankowski JJ, Van Rossem R. 2005. Pathways from prematurity and infant abilities to later cognition. Child Development. 76:1172–1184. 16274433 10.1111/j.1467-8624.2005.00843.x

[ref68] Rosenberg KR . 1992. The evolution of modern human childbirth. American Journal of Physical Anthropology. 35:89–124.

[ref69] Rutherford S , Sturmfels P, Angstadt M, Hect J, Wiens J, van den Heuval MI, Scheinost D, Thomason M, Sripada C. 2019. Observing the origins of human brain development: automated processing of fetal fMRI. bioRxiv. 525386.

[ref70] Santos A , Mier D, Kirsch P, Meyer-Lindenberg A. 2011. Evidence for a general face salience signal in human amygdala. NeuroImage. 54:3111–3116. 21081170 10.1016/j.neuroimage.2010.11.024

[ref71] Schöpf V , Kasprian G, Brugger P, Prayer D. 2012. Watching the fetal brain at ‘rest’. International Journal of Developmental Neuroscience. 30:11–17. 22044604 10.1016/j.ijdevneu.2011.10.006

[ref72] Seeley WW , Menon V, Schatzberg AF, Keller J, Glover GH, Kenna H, Reiss AL, Greicius MD. 2007. Dissociable intrinsic connectivity networks for salience processing and executive control. Journal of Neuroscience. 27:2349–2356. 17329432 10.1523/JNEUROSCI.5587-06.2007 PMC2680293

[ref73] Senju A. 2012. Spontaneous theory of mind and its absence in autism spectrum disorders. The Neuroscientist. 18:108–113. 21609942 10.1177/1073858410397208 PMC3796729

[ref74] Simon HA . 1986. The Role of Attention in Cognition. New York, NY: Academic Press.

[ref75] Squire LR , Zola-Morgan S. 1991. The medial temporal lobe memory system. Science. 253:1380–1386. 1896849 10.1126/science.1896849

[ref76] Thomason ME , Dassanayake MT, Shen S, Katkuri Y, Alexis M, Anderson AL, Yeo L, Mody S, Hernandez-Andrade E, Hassan SS. 2013. Cross-hemispheric functional connectivity in the human fetal brain. Science Translational Medicine. 5:173ra124. 10.1126/scitranslmed.3004978 PMC3618956 23427244

[ref77] Thomason ME , Brown JA, Dassanayake MT, Shastri R, Marusak HA, Hernandez-Andrade E, Yeo L, Mody S, Berman S, Hassan SS. 2014. Intrinsic functional brain architecture derived from graph theoretical analysis in the human fetus. PLoS One. 9:e94423. 24788455 10.1371/journal.pone.0094423 PMC4006774

[ref78] Thomason ME , Grove LE, Lozon TA Jr, Vila AM, Ye Y, Nye MJ, Manning JH, Pappas A, Hernandez-Andrade E, Yeo L. 2015. Age-related increases in long-range connectivity in fetal functional neural connectivity networks in utero. Developmental Cognitive Neuroscience. 11:96–104. 25284273 10.1016/j.dcn.2014.09.001 PMC4532276

[ref79] Thomason ME , Scheinost D, Manning JH, Grove LE, Hect J, Marshall N, Hernandez-Andrade E, Berman S, Pappas A, Yeo L. 2017. Weak functional connectivity in the human fetal brain prior to preterm birth. Scientific Reports. 7:1–10. 28067865 10.1038/srep39286 PMC5221666

[ref80] Thomason ME , Hect J, Waller R, Manning JH, Stacks AM, Beeghly M, Boeve JL, Wong K, Van Den Heuvel MI, Hernandez-Andrade E. 2018. Prenatal neural origins of infant motor development: associations between fetal brain and infant motor development. Development and Psychopathology. 30:763–772. 30068433 10.1017/S095457941800072X PMC6261435

[ref81] Turati C , Simion F. 2002. Newborns’ recognition of changing and unchanging aspects of schematic faces. Journal of Experimental Child Psychology. 83:239–261. 12470960 10.1016/s0022-0965(02)00148-0

[ref82] Turk E , van den Heuvel MI, Benders MJ, de Heus R, Franx A, Manning JH, Hect JL, Hernandez-Andrade E, Hassan SS, Romero R. 2019. Functional connectome of the fetal brain. Journal of Neuroscience. 39:9716–9724. 31685648 10.1523/JNEUROSCI.2891-18.2019 PMC6891066

[ref83] Tye C , Mercure E, Ashwood KL, Azadi B, Asherson P, Johnson MH, Bolton P, McLoughlin G. 2013. Neurophysiological responses to faces and gaze direction differentiate children with ASD, ADHD and ASD+ ADHD. Developmental Cognitive Neuroscience. 5:71–85. 23466656 10.1016/j.dcn.2013.01.001 PMC6987819

[ref84] Ungless MA . 2004. Dopamine: the salient issue. Trends in Neurosciences. 27:702–706. 15541509 10.1016/j.tins.2004.10.001

[ref85] van den Heuvel MI , Thomason ME. 2016. Functional connectivity of the human brain in utero. Trends in Cognitive Sciences. 20:931–939. 27825537 10.1016/j.tics.2016.10.001 PMC5339022

[ref86] van den Heuvel MI , Turk E, Manning JH, Hect J, Hernandez-Andrade E, Hassan SS, Romero R, van den Heuvel MP, Thomason ME. 2018. Hubs in the human fetal brain network. Developmental Cognitive Neuroscience. 30:108–115. 29448128 10.1016/j.dcn.2018.02.001 PMC5963507

[ref87] Van Dijk KR , Hedden T, Venkataraman A, Evans KC, Lazar SW, Buckner RL. 2009. Intrinsic functional connectivity as a tool for human connectomics: theory, properties, and optimization. Journal of Neurophysiology. 103:297–321. 19889849 10.1152/jn.00783.2009 PMC2807224

[ref88] Vann SD , Nelson AJ. 2015. The mammillary bodies and memory: more than a hippocampal relay. In Progress in brain research Amsterdam: Elsevier p. 163–185. 10.1016/bs.pbr.2015.03.006 PMC4498492 26072239

[ref89] Varendi H , Porter R. 2001. Breast odour as the only maternal stimulus elicits crawling towards the odour source. Acta Paediatrica. 90:372–375. 11332925

[ref90] Vázquez-Rodríguez B , Suárez LE, Markello RD, Shafiei G, Paquola C, Hagmann P, Van Den Heuvel MP, Bernhardt BC, Spreng RN, Misic B. 2019. Gradients of structure–function tethering across neocortex. Proceedings of the National Academy of Sciences. 116:21219–21227. 10.1073/pnas.1903403116 PMC6800358 31570622

[ref91] Wheelock M , Hect J, Hernandez-Andrade E, Hassan S, Romero R, Eggebrecht A, Thomason M. 2019. Sex differences in functional connectivity during fetal brain development. Developmental Cognitive Neuroscience. 36:100632. 30901622 10.1016/j.dcn.2019.100632 PMC6944279

[ref92] Whitfield-Gabrieli S , Nieto-Castanon A. 2012. Conn: a functional connectivity toolbox for correlated and anticorrelated brain networks. Brain Connectivity. 2:125–141. 22642651 10.1089/brain.2012.0073

[ref93] Winton-Brown TT , Fusar-Poli P, Ungless MA, Howes OD. 2014. Dopaminergic basis of salience dysregulation in psychosis. Trends in Neurosciences. 37:85–94. 24388426 10.1016/j.tins.2013.11.003

