# Supplementary material for: Subcortico-Cortical Functional Connectivity in the Fetal Brain: A Cognitive Development Blueprint
Source: Cereb Cortex Commun. 2020 Apr 3;1(1):tgaa008. doi: 10.1093/texcom/tgaa008 (PMC8152909; doi:10.1093/texcom/tgaa008)
Supplement: SOM_Canini_Final_CCC_tgaa008 [file som_canini_final_ccc_tgaa008.docx]

# Subcortico-Cortical Functional Connectivity in the Fetal Brain: A Cognitive Development Blueprint

# Supplementary Online Material

# 1 Image Processing Detail

The choice to acquire multiple, short lasting rs-fMRI sessions (over a single, long-lasting one) was related to our goal to minimize movement-related variability in our dataset. Undetected motion has been reported to affect observed rs-fMRI functional connectivity patterns (Power et al., 2012) and, while being an issue of general concern for rs-fMRI, it is of particular relevance when concentrating on fetal functional datasets. Periods of stillness interleaved with periods of intense movement can characterize fetal brain functional activity inside the mother’s womb. To this end acquiring multiple short-lasting scans allowed us to evaluate and discard sessions suffering from high rates of motion in real time and to (eventually) acquire a vicarious session at the end of the motion period, with a relatively low cost in terms of both data (max 60 volumes) and scanning time (max 2 mins) loss. Critically, this also allowed us to maximize the quality of raw data input to preprocessing, with gross movement discarded *a priori*, i.e. during acquisition itself. Specifically, we were able to detect and scrub motion from our dataset in a hierarchical fashion, by taking into account signal variability changes emerging at both the within and between sessions levels. Details on this within-between sessions processing gradient are provided in the following paragraphs.
*Within-Session (WS) Processing : Orientation Matching* First, all volumes were manually oriented and realigned to a session-specific functional reference volume using SPM12 (<https://www.fil.ion.ucl.ac.uk/spm/>). In order to maximize alignment results, a volume was deemed eligible as functional reference of its session only if (i) showing no major artifacts in terms of signal homogeneity (as detectable by visual inspection of the timeseries) and (ii) showing a consistent orientation overlap with all the other volumes in the timeseries.
*Within-Session (WS) Processing : Scrub of Outlier Volumes* Realigned functional volumes were then inspected for outliers at the WS level, using a two-step outliers detection approach. Specifically, we aimed at identifying both (i) smaller, derivative movements occurring slowly over time (best detected by frame-to-mean displacement analyses) and (ii) larger movements occurring suddenly (best detected by frame-to-frame displacement analyses). Frame-to-mean displacement was estimated using the artifact detection toolbox (ART) (<https://www.nitrc.org/projects/artifact_detect>) with volumes considered outliers if showing a global signal intensity variation exceeding 1.5 STD (with respect to mean global intensity) and/or if showing motion greater than 2mm in any direction.
Frame-to-frame estimates of motion (FD, Power et al., 2012, 2014) and signal intensity (DVARS, Smyser et al., 2010) changes were calculated using the motion outliers detection algorithms provided with FSL (<https://fsl.fmrib.ox.ac.uk/fsl/fslwiki/FSLMotionOutliers>). Scans were deemed outliers if falling over the 75th percentile (plus 1.5 times the interquartile range) of one or both parameters distributions. Output from both ART and DVARS/FD diagnostic procedures were merged to produce a combined frame-to-mean/frame-to-frame index, based on which outliers were scrubbed from the volumes sample included in further preprocessing steps.
*Within-Session (WS) Processing : Maternal Abdominal Tissue Removal* As a final, WS processing step a spherical mask was drawn on the functional reference and applied to all volumes in order to exclude maternal abdominal tissue (MAT) (see Figure 1 SOM). MAT exclusion was primarily aimed at partialling-out a major source of signal variability from our dataset, in order to maximize the effectiveness of between-sessions orientation procedures.
*Between-Sessions (BS) Processing: Realignment and Scrubbing* Volumes surviving the WS outliers detection step were then concatenated into a single, continuous session and realigned to a common functional reference (calculated as the average of all session-specific functional references). Realigned volumes were then inspected for BS outliers using the same procedures used for WS outliers assessment (see above). Outlier volumes were scrubbed from the dataset and excluded from further processing steps while volumes surviving the outlier detection procedure were further realigned to the mean functional reference, in order to maximize realignment between the volumes included in the final rs-fMRI dataset of each subject.
*Fetal functional spatial normalization* In order to allow for group-level statistics, functional volumes were normalized into a standardized space. Given the GW range of our sample we used an ad hoc approach aimed at i) optimizing skull- and body- stripping of the fetal EPI images in order to minimize the influence of the surrounding tissues on brain spatial normalization of the TS, without losing any inner-brain voxels; and ii) increasing calculation accuracy of the deformation parameters to a fetal brain template generated for each subject and used to transform each realigned and stripped functional volume into a common 28 GW fetal template space (i.e. group median) for group-level analysis.
The optimized stripping of fetal functional images for spatial normalization (i) was performed with the “New Segment” function in SPM, used to create an inner brain mask on the between time-series reference EPI image in the subject’s native space, based on different fetal tissue class probability maps (i.e. 1 - cortical plate and cerebellum, 2 - white matter (WM), 3 - cerebro-spinal fluid (CSF), 4 - deep grey matter (DGM), 5 - hippocampus, 6 - amygdala, 7 - brainstem). The resulting inner-brain mask image was used to mask out residual surrounding tissue on pre-processed EPI brain volume, in turn producing an optimally stripped fetal functional time-series.
The transformation and forward deformation parameters to the 28 GW fetal template brain (Gholipour et al., 2017) (ii) were created for each participant, as outputs of the “New Segment” step. These parameters were used to transform each stripped functional image into the 28 GW fetal template space for group-level connectivity analysis, optimizing spatial normalization accuracy into a common fetal reference space for each subject. This stripped and normalized functional dataset was finally Gaussian smoothed (FWHM = 4 mm).


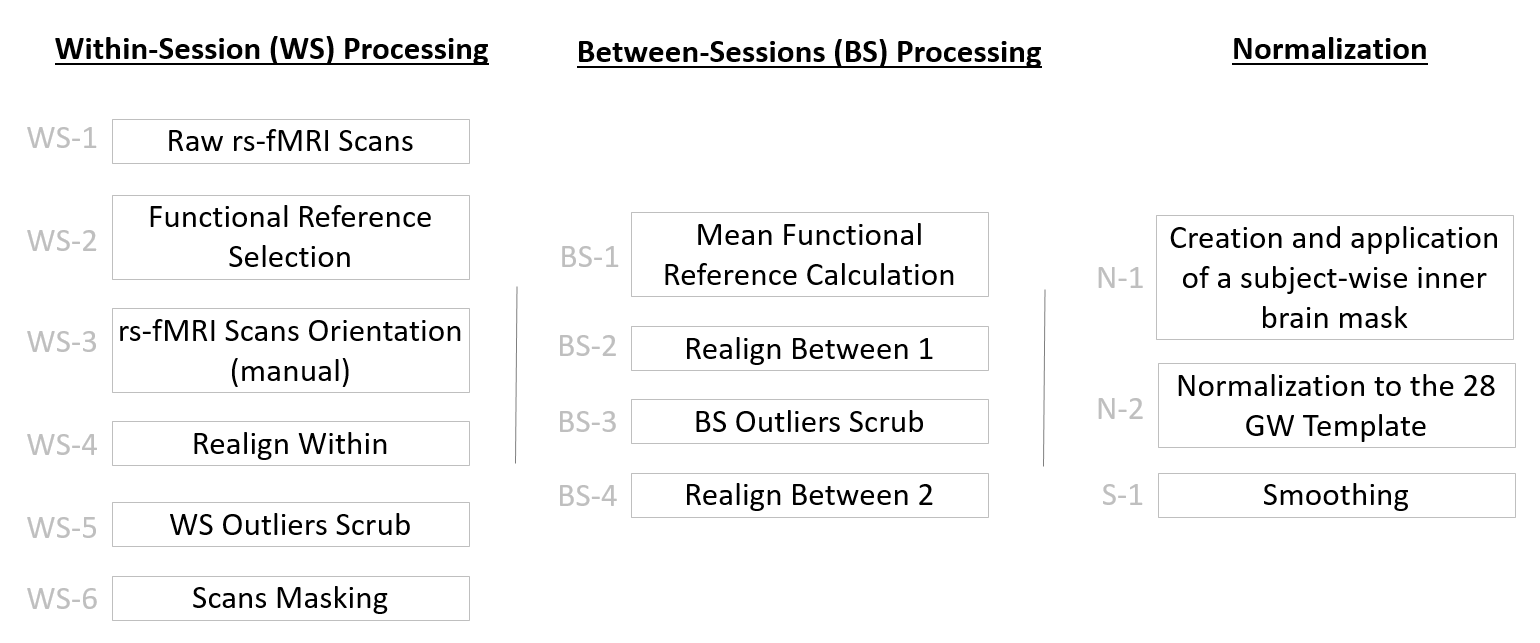


**Figure 1 SOM** Pipeline employed for preprocessing of the rs-fMRI timeseries (TS). Fetal functional volumes are first aligned, diagnosed for outliers and scrubbed on a session-specific basis (WS, left panel). The same alignement and diagnostics are then applied to unscrubbed volumes in order to investigate signal variability between sessions (BS, central panel). Surviving volumes are finally normalized to a standardized space and smoothed for group-level analysis.

# 2 Statistical Analysis Detail

*Seed-Based-Connectivity Analysis*
*1^st^ Level Analysis:* The CONN functional connectivity toolbox (Whitfield-Gabrieli and Nieto Castanon, 2012, <https://web.conn-toolbox.org/>) ver. 18.b was used to estimate connectivity patterns between the selected subcortical nuclei and the whole brain. Residual motion was also regressed out by including session-specific DVARS and FD estimates as covariates of no interest. Seed regions were defined using the Gholipour 28 GW fetal brain anatomical parcellation (Gholipour et al., 2017), to which all volumes were previously normalized (see above).
 *2^nd^* *Level Analysis : Unconstrained Connectivity*  Connectivity Maps resulting from 1^st^ level analysis were then submitted to 2^nd^ level, random effects modeling and tested for patterns of significant increased connectivity between each seed and the whole brain, using a one sample t-test model. To this end smoothed, normalized rs-fMRI volumes were entered as dependent variable and a t-contrast was specified to test for the positive component of group-related effects.
*2^nd^* *Level Analysis : Connectivity Patterns as a Function of GW*  The same model was also tested for the effects of gestational week on connectivity. To this end a covariate coding for GW at scan of each subject was specified and tested for any positive effect on connectivity patterns increase.
*Results Exploration* Results were investigated using a combined Family Wise Error/False Discovery Rate (FWE/FDR) thresholding approach. First, clusters of connectivity were deemed significant only if surviving a p = .05 FWE correction at the voxel level. Second, the reliability of the observed clusters was further tested by exploring results using an FDR correction constrained to p = .001, such that the resulting clusters had a probability of 0.01% to be false positive observations. As a result only voxels with a low probability of resulting in a false positive activation (at the individual voxel level) or a false discovery within a group of voxels (with respect to the overall pattern of observed results) were considered for discussion.
We believe such a thresholding approach as being the most suitable for setting robust inference on functional fetal statistical maps results. Evaluating results without any spatial constraint (i.e. using FDR correction only) in such a context of high spatial proximity between neighboring structures, could have resulted in the emergence of broad patterns of connectivity, with a substantial lack of power in the identification of areas where activity reaches its peak (i.e. with a single, broad cluster including all peaking subclusters). At the same time, relying exclusively on a FWE correction in such a limited brain volume could have led to an extreme focus on clusters showing peak values, with a potential loss in our power to observe more broad patterns of connectivity which, given the developmental nature of our sample, are instead likely to be observed.

3 Clusters Localization Consistency Across the 25-32 GW Range
Clusters of significant seed-to-voxel correlations reported in Table 1 of the main manuscript were localized on the 28 GW template space, to which all EPI volumes were normalized for 1st and 2nd level analyses. Nevertheless, given the rapidly evolving nature of transient cortical structures during the gestational week range included in our sample (i.e. 25-32 GW), localization consistency was further investigated. To this end i) the 25, 27, 30 and 32 template spaces (Gholipour et al., 2017) were chosen as representative timepoints (i.e. 1st, 25th, 75th and 100th centiles) of the sample GW distribution, ii) thresholded seed-to-voxel connectivity maps were normalized from their common 28 GW space to the 25, 27 GW (backward deformations) and 30, 32 GW (forward deformations) template spaces and iii) labeled with a GW-specific localization on transient layers structures (i.e. from the inner to the outermost layer: Ventricular Zone -VZ; Intermediate Zone - IZ; Subplate - SP; Cortical Plate - CP). iv) Localization consistency was assessed as the probability of a given cluster to fall within the same transient cortical layer across the different GW timepoints taken into consideration. The transient-layer consistency accuracy of clusters falling into VZ, IZ and SP transient layers was calculated as the probability (expressed in percentage) of each layer to host a certain cluster at the 25, 27, 28 and 30 GW timepoints. The 32nd GW parcellation was excluded from the transient-layer consistency calculation, due to full maturation of the CP at this time in gestation. Localization consistency of the permanent CP was instead calculated based on all the GW taken into consideration (i.e. 25, 27, 28, 30 and 32 GW). Both transient-layer and CP localization consistency results are reported in the Table 3 of the SOM below. Furthermore Figure 2 (SOM, below) shows seeds (i.e. bilateral Thalamus, bilateral Hippocampus, bilateral Caudate Nucleus, bilateral Amygdala, bilateral Lentiform Nucleus, bilateral Subthalamic Nucleus) at the 25, 27, 28, 30 and 32 GW timepoints, for a visual inspection of subcortical structures maturation consistency across the considered GW range.

4 Biometrical Consistency
We investigated biometrical consistency between each subject’s Biparietal and Fronto-Occipital diameters (BPD and FOD, respectively) and normative measures (Garel, 2004) at 28 weeks of gestation. To this end BPD and FOD of each subject were estimated by a specialized neuroradiologist, on a T2 structural scan. Specifically BPD was estimated on the coronal plane, while FOD was estimated on the sagittal plane. The entire sample fell into the 28 GW ranges, for both BPD (52-69mm) and FOD (70-90mm) measurements. Subject-specific information on BPD and FOD are provided in Table 4 below.

# Figure 2 SOM : Subcortical Seeds Maturation across the 25-32 GW range


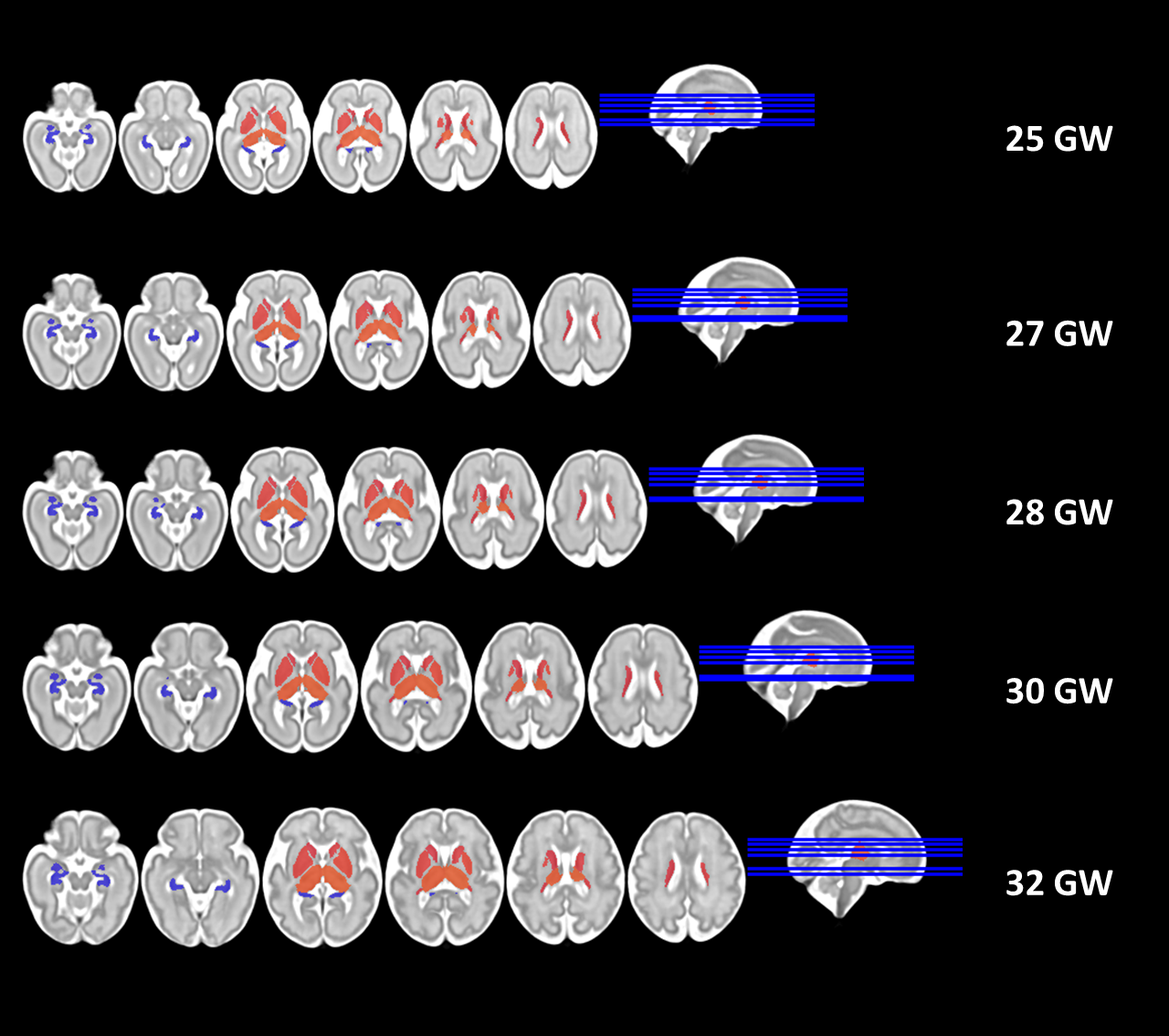

**Figure 2 SOM** Subcortical seeds maturation at the 25, 27, 28, 30 and 32 GW timepoints (i.e. 1st, 25th, 50th, 75th and 100th centiles of the GW distribution included in the sample), shown on the anatomical templates provided by Gholipour and colleagues (2017).

# Table 1 : Sample Characteristics Detail


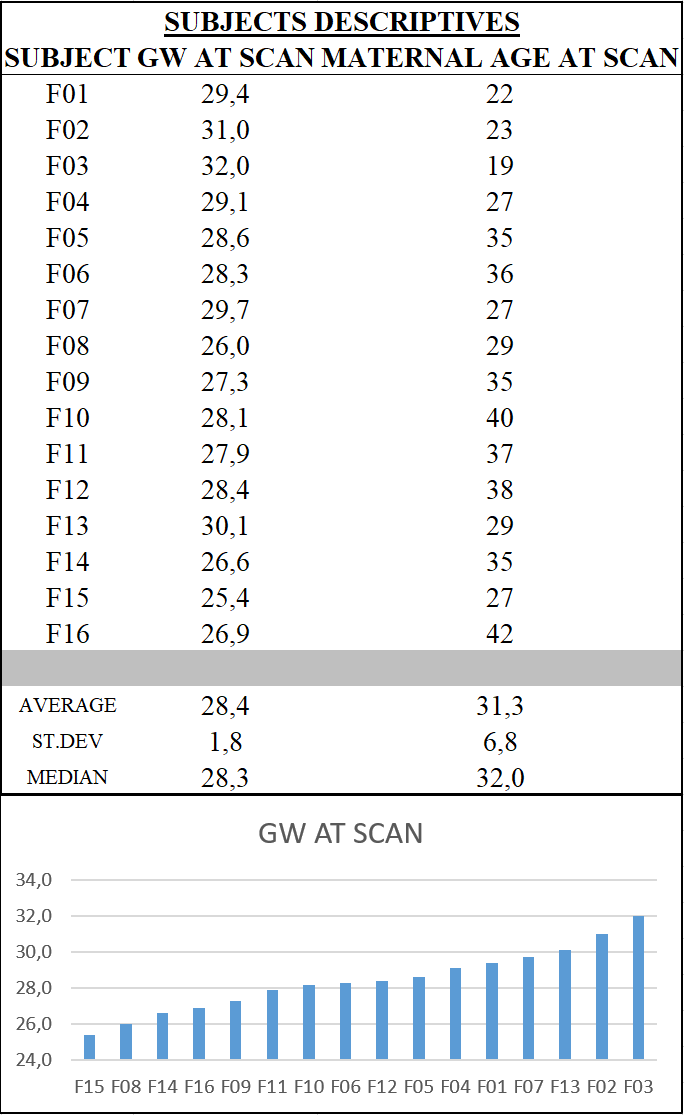


**Table 1 SOM** Upper Panel: information on Gestational Week (GW) and Age at scan is provided for each subject included in the analysis. Lower Panel: plot of the neurodevelopmental window investigated in this study, ranging from the 25^th^ to the 32^nd^ GW. GW are expressed in ‘week,days’ format, with days converted on a 0-1 scale (with 0,1 = 1 day; 0,3 = 2 days; 0,4 = 3 days; 0,6 = 4 days; 0,7 = 5 days; 0,9 = 6 days).

# Table 2 : Data Acquisition and Scrubbing Detail


**Table 2 SOM** Resting State fMRI signal analyzed in the study. Details are provided for both i) the total number of rs-fMRI sessions and volumes acquired and selected to enter the preprocessing phase (left panel) and ii) the total number (along with percentage) of volumes and corresponding minutes of signal included in the analysis after scrubbing (right panel).

Table 3 : Clusters Localization Consistency

**Table 3 SOM** Cortical clusters localization consistency across the experimental GW range included in the study. For each cluster the cortical domain is reported along with layer-specific localization at the 25, 27, 28, 30 and 32 GW timepoints, corresponding to the 1^st^, 25^th^, 50^th^, 75^th^ and 100^th^ centiles of the GW distribution. For each GW layers hosting a connectivity cluster are shown in blue, while red labeled cells indicate a layer not hosting connectivity. Grey cells indicate the absence of transient layers for the 32 GW, a time in gestation when connectivity is fully hosted by the permanent CP. Localization consistency (LC) is provided as the overall probability (expressed in percentage) for a cluster to be hosted by a certain layer across GWs (last column). LC for the SP, IZ and SP transient layers is calculated on the 25, 27, 28 and 30 GW timepoints, while CP localization consistency is calculated on all GW timepoints.

Table 4 Biometrical Consistency


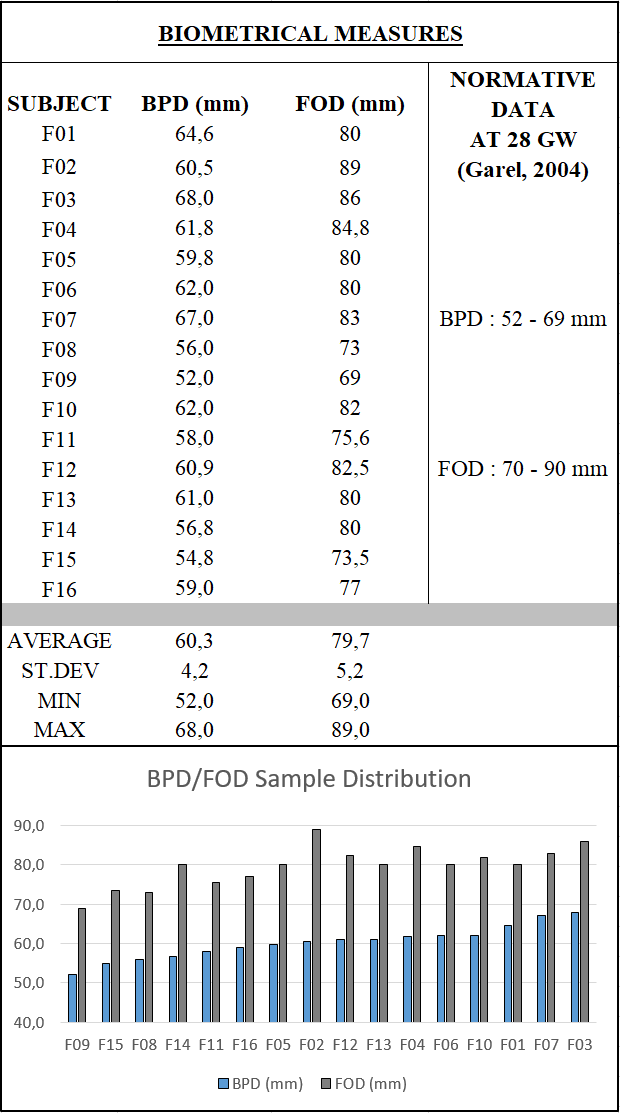


**Table 4 SOM** Information on Biparietal (BPD) and Fronto-Occipital (FOD) diameters is provided for each subject included in the study, along with normative data (Garel, 2004) for the 28 GW anatomy, to which all subjects were normalized for analysis (upper panel). Sample BPD and FOD distributions are also shown in the lower panel.

References

- Garel C, 2004. MRI of the Fetal Brain: Springer.
- Gholipour A, Rollins CK, Velasco-Annis C, Ouaalam A, Akhondi-Asl A, Afacan O, Ortinau CM, Clancy S, Limperopoulos C, Yang E. 2017. A normative spatiotemporal MRI atlas of the fetal brain for automatic segmentation and analysis of early brain growth. Scientific reports. 7:476.
- Power JD, Barnes KA, Snyder AZ, Schlaggar BL, Petersen SE. 2012. Spurious but systematic correlations in functional connectivity MRI networks arise from subject motion. Neuroimage. 59:2142-2154.
- Power JD, Mitra A, Laumann TO, Snyder AZ, Schlaggar BL, Petersen SE. 2014. Methods to detect, characterize, and remove motion artifact in resting state fMRI. Neuroimage. 84:320-341.
- Whitfield-Gabrieli S, Nieto-Castanon A. 2012. Conn: a functional connectivity toolbox for correlated and anticorrelated brain networks. Brain connectivity. 2:125-141.
